# Dichloridobis(isoquinoline-κ*N*)zinc(II)

**DOI:** 10.1107/S1600536810024803

**Published:** 2010-07-03

**Authors:** Meng-Jiao Li, Jing-Jing Nie, Duan-Jun Xu

**Affiliations:** a Department of Chemistry, Zhejiang University, People’s Republic of China

## Abstract

In the title compound, [ZnCl_2_(C_9_H_7_N)_2_], the Zn^II^ cation is coordinated by two Cl^−^ anions and two isoquinoline ligands in a distorted ZnCl_2_N_2_ tetra­hedral geometry; the two isoquinoline ring systems are twisted with respect to each other at a dihedral angle of 45.72 (8)°. The parallel isoqiunoline ring systems of adjacent mol­ecules are partially overlapped, with the shorter face-to-face distance of 3.438 (19) Å indicating the existence of weak π–π stacking in the crystal structure.

## Related literature

For general background to π-π stacking, see: Deisenhofer & Michel (1989 ▶); Su & Xu (2004 ▶); Xu *et al.* (2007 ▶). For π-π stacking between isoquinoline ring systems in a Co^II^ complex, see: Li *et al.* (2010 ▶).
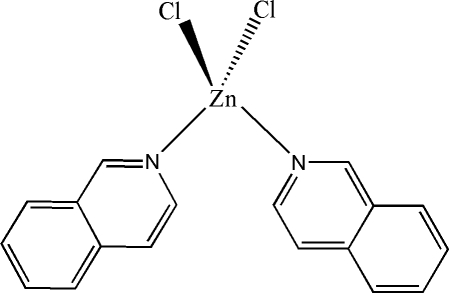


## Experimental

### 

#### Crystal data

[ZnCl_2_(C_9_H_7_N)_2_]
*M* *_r_* = 394.58
Monoclinic, 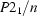

*a* = 7.8956 (15) Å
*b* = 13.363 (2) Å
*c* = 15.677 (2) Å
β = 90.220 (8)°
*V* = 1654.0 (5) Å^3^
*Z* = 4
Mo *K*α radiation
μ = 1.81 mm^−1^
*T* = 294 K
0.40 × 0.32 × 0.30 mm

#### Data collection

Rigaku R-AXIS RAPID IP diffractometer
Absorption correction: multi-scan (*ABSCOR*; Higashi, 1995 ▶) *T*_min_ = 0.788, *T*_max_ = 0.862
11270 measured reflections
2975 independent reflections
1933 reflections with *I* > 2σ(*I*)
*R*_int_ = 0.032

#### Refinement

*R*[*F* ^2^ > 2σ(*F* ^2^)] = 0.056
*wR*(*F* ^2^) = 0.153
*S* = 0.95
2975 reflections
208 parameters
H-atom parameters constrained
Δρ_max_ = 1.33 e Å^−3^
Δρ_min_ = −0.39 e Å^−3^

### 

Data collection: *PROCESS-AUTO* (Rigaku, 1998 ▶); cell refinement: *PROCESS-AUTO*; data reduction: *CrystalStructure* (Rigaku/MSC, 2002 ▶); program(s) used to solve structure: *SIR92* (Altomare *et al.*, 1993 ▶); program(s) used to refine structure: *SHELXL97* (Sheldrick, 2008 ▶); molecular graphics: *ORTEP-3* (Farrugia, 1997 ▶); software used to prepare material for publication: *WinGX* (Farrugia, 1999 ▶) and *PLATON* (Spek, 2009 ▶).

## Supplementary Material

Crystal structure: contains datablocks I, global. DOI: 10.1107/S1600536810024803/hb5517sup1.cif

Structure factors: contains datablocks I. DOI: 10.1107/S1600536810024803/hb5517Isup2.hkl

Additional supplementary materials:  crystallographic information; 3D view; checkCIF report

## Figures and Tables

**Table 1:** Selected bond lengths (Å)

Zn—N1	2.062 (4)
Zn—N2	2.052 (4)
Zn—Cl1	2.2235 (13)
Zn—Cl2	2.2262 (13)

**Fig. 1.**
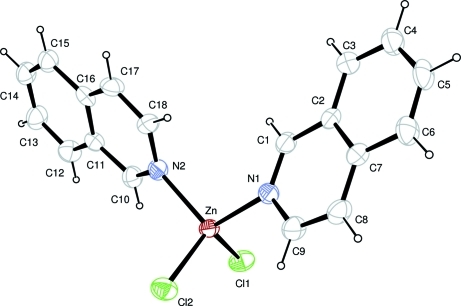
The molecular structure of (I) with 50% probability displacement ellipsoids (arbitrary spheres for H atoms).

**Fig. 2.**
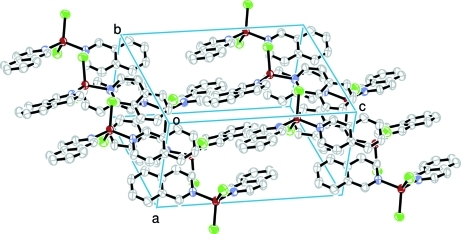
The unit cell packing diagram of (I) showing the parallel arrangement of isoquinoline ligands. H atoms have been omitted for clarity.

## supplementary crystallographic information

### Comment

The π-π stacking between aromatic rings is an important non-covalent interaction and correlated with the electron transfer process in some biological systems (Deisenhofer & Michel, 1989). As part of our ongoing investigation on the nature of π-π stacking (Su & Xu, 2004; Xu *et al.*, 2007), the title complex incorporating isoquinoline ligand has recently been prepared in the laboratory and its crystal structure is reported here.

In the title compound, the Zn cation is coordinated by two Cl^-^ anions and two isoquinoline ligands in a distorted ZnCl_2_N_2_ tetrahedral geometry (Fig. 1). The two isoquinoline ring systems are tweisted to each other at a dihedral angle of 45.72 (8)°. The parallel N2-isoqiunoline and N2^i^-isoquinoline ring systems [symmetry code: (i) 2 - *x*, 1 - *y*,1 - *z*] of adjacent molecules are partially overlapped, the shorter face-to-face distance of 3.438 (19) Å indicates the existence of weak π-π stacking in the crystal structure (Fig. 2), similar to that found in a polymeric Co complex with isoquinoline ligands (Li *et al.* 2010). No hydrigen bonding is present in the crystal structure.

### Experimental

Isoquinoline (0.23 ml, 2 mmol) and ZnCl_2_ (0.14 g, 1 mmol) were dissolved in an absolute ethanol (10 ml). The solution was refluxed for 12 h. After cooling to room temperature, the solution was filtered and colourless prisms of (I) were obtained from the filtrate after 2 d.

### Refinement

H atoms were placed in calculated positions with C—H = 0.93 (aromatic) and refined in riding mode with *U*_iso_(H) = 1.2*U*_eq_(C). An ADDSYM-XCT check (Spek, 2009) shows no additional symmetry for the structure. An attempt at refinement with higher symmetry [orthorhombic Pmn21] did not give a reasonable solution.

### Crystal data

**Table:**

[ZnCl_2_(C_9_H_7_N)_2_]	*F*(000) = 800
*M**_r_* = 394.58	*D*_x_ = 1.585 Mg m^−^^3^
Monoclinic, *P*2_1_/*n*	Mo *K*α radiation, λ = 0.71073 Å
Hall symbol: -P 2yn	Cell parameters from 6266 reflections
*a* = 7.8956 (15) Å	θ = 3.3–24.6°
*b* = 13.363 (2) Å	µ = 1.81 mm^−^^1^
*c* = 15.677 (2) Å	*T* = 294 K
β = 90.220 (8)°	Prism, colorless
*V* = 1654.0 (5) Å^3^	0.40 × 0.32 × 0.30 mm
*Z* = 4	

### Data collection

**Table:**

Rigaku R-AXIS RAPID IP diffractometer	2975 independent reflections
Radiation source: fine-focus sealed tube	1933 reflections with *I* > 2σ(*I*)
graphite	*R*_int_ = 0.032
Detector resolution: 10.0 pixels mm^-1^	θ_max_ = 25.2°, θ_min_ = 3.3°
ω scans	*h* = −9→9
Absorption correction: multi-scan (*ABSCOR*; Higashi, 1995)	*k* = −16→15
*T*_min_ = 0.788, *T*_max_ = 0.862	*l* = −18→17
11270 measured reflections	

### Refinement

**Table:**

Refinement on *F*^2^	Primary atom site location: structure-invariant direct methods
Least-squares matrix: full	Secondary atom site location: difference Fourier map
*R*[*F*^2^ > 2σ(*F*^2^)] = 0.056	Hydrogen site location: inferred from neighbouring sites
*wR*(*F*^2^) = 0.153	H-atom parameters constrained
*S* = 0.95	*w* = 1/[σ^2^(*F*_o_^2^) + (0.1036*P*)^2^] where *P* = (*F*_o_^2^ + 2*F*_c_^2^)/3
2975 reflections	(Δ/σ)_max_ < 0.001
208 parameters	Δρ_max_ = 1.33 e Å^−^^3^
0 restraints	Δρ_min_ = −0.39 e Å^−^^3^

### Special details

**Table:**

Geometry. All e.s.d.'s (except the e.s.d. in the dihedral angle between two l.s. planes) are estimated using the full covariance matrix. The cell e.s.d.'s are taken into account individually in the estimation of e.s.d.'s in distances, angles and torsion angles; correlations between e.s.d.'s in cell parameters are only used when they are defined by crystal symmetry. An approximate (isotropic) treatment of cell e.s.d.'s is used for estimating e.s.d.'s involving l.s. planes.
Refinement. Refinement of *F*^2^ against ALL reflections. The weighted *R*-factor *wR* and goodness of fit *S* are based on *F*^2^, conventional *R*-factors *R* are based on *F*, with *F* set to zero for negative *F*^2^. The threshold expression of *F*^2^ > σ(*F*^2^) is used only for calculating *R*-factors(gt) *etc*. and is not relevant to the choice of reflections for refinement. *R*-factors based on *F*^2^ are statistically about twice as large as those based on *F*, and *R*- factors based on ALL data will be even larger.

### Fractional atomic coordinates and isotropic or equivalent isotropic displacement parameters (Å^2^)

**Table:**

	*x*	*y*	*z*	*U*_iso_*/*U*_eq_	
Zn	0.75125 (6)	0.30754 (4)	0.25278 (3)	0.0408 (2)	
Cl1	0.51169 (15)	0.22275 (10)	0.27066 (8)	0.0597 (4)	
Cl2	0.98654 (16)	0.21580 (10)	0.25609 (9)	0.0616 (4)	
N1	0.7603 (4)	0.3828 (3)	0.1381 (2)	0.0448 (9)	
N2	0.7615 (5)	0.4115 (3)	0.3490 (2)	0.0466 (9)	
C1	0.7034 (6)	0.4723 (4)	0.1236 (3)	0.0482 (11)	
H1	0.6550	0.5071	0.1688	0.058*	
C2	0.7109 (5)	0.5212 (3)	0.0418 (3)	0.0423 (10)	
C3	0.6513 (6)	0.6175 (4)	0.0286 (3)	0.0590 (13)	
H3	0.6042	0.6540	0.0731	0.071*	
C4	0.6632 (7)	0.6570 (4)	−0.0501 (3)	0.0651 (14)	
H4	0.6234	0.7216	−0.0595	0.078*	
C5	0.7340 (7)	0.6037 (5)	−0.1187 (3)	0.0645 (15)	
H5	0.7409	0.6337	−0.1721	0.077*	
C6	0.7913 (7)	0.5106 (5)	−0.1080 (3)	0.0620 (14)	
H6	0.8365	0.4755	−0.1538	0.074*	
C7	0.7822 (6)	0.4647 (4)	−0.0243 (3)	0.0481 (12)	
C8	0.8407 (6)	0.3682 (4)	−0.0097 (3)	0.0589 (13)	
H8	0.8863	0.3301	−0.0536	0.071*	
C9	0.8294 (6)	0.3310 (4)	0.0713 (3)	0.0559 (12)	
H9	0.8708	0.2670	0.0816	0.067*	
C10	0.7032 (6)	0.3890 (4)	0.4234 (3)	0.0525 (12)	
H10	0.6485	0.3279	0.4303	0.063*	
C11	0.7191 (5)	0.4547 (3)	0.4968 (3)	0.0444 (11)	
C12	0.6585 (7)	0.4275 (4)	0.5760 (3)	0.0620 (14)	
H12	0.6066	0.3658	0.5846	0.074*	
C13	0.6774 (7)	0.4941 (5)	0.6412 (3)	0.0676 (15)	
H13	0.6375	0.4770	0.6950	0.081*	
C14	0.7542 (6)	0.5864 (4)	0.6299 (3)	0.0591 (14)	
H14	0.7638	0.6297	0.6761	0.071*	
C15	0.8152 (6)	0.6148 (4)	0.5537 (3)	0.0582 (13)	
H15	0.8673	0.6767	0.5473	0.070*	
C16	0.7987 (5)	0.5473 (3)	0.4815 (3)	0.0448 (11)	
C17	0.8591 (6)	0.5706 (4)	0.4020 (3)	0.0552 (13)	
H17	0.9120	0.6316	0.3920	0.066*	
C18	0.8402 (6)	0.5027 (4)	0.3381 (3)	0.0551 (13)	
H18	0.8824	0.5185	0.2845	0.066*	

### Atomic displacement parameters (Å^2^)

**Table:**

	*U*^11^	*U*^22^	*U*^33^	*U*^12^	*U*^13^	*U*^23^
Zn	0.0544 (4)	0.0332 (3)	0.0347 (3)	0.0000 (2)	0.0035 (2)	−0.0002 (2)
Cl1	0.0625 (8)	0.0496 (8)	0.0672 (8)	−0.0098 (6)	0.0145 (6)	−0.0045 (6)
Cl2	0.0638 (8)	0.0546 (8)	0.0665 (8)	0.0131 (6)	0.0068 (6)	−0.0011 (6)
N1	0.051 (2)	0.042 (2)	0.042 (2)	−0.0036 (18)	−0.0006 (16)	0.0036 (17)
N2	0.053 (2)	0.049 (2)	0.037 (2)	0.0060 (19)	0.0019 (16)	−0.0027 (18)
C1	0.053 (3)	0.048 (3)	0.043 (3)	−0.004 (2)	−0.002 (2)	−0.006 (2)
C2	0.047 (2)	0.043 (3)	0.037 (2)	−0.007 (2)	−0.0001 (19)	−0.007 (2)
C3	0.072 (3)	0.050 (3)	0.055 (3)	0.004 (3)	−0.007 (2)	−0.003 (3)
C4	0.073 (3)	0.070 (4)	0.053 (3)	−0.011 (3)	−0.010 (3)	0.012 (3)
C5	0.077 (4)	0.082 (4)	0.034 (3)	−0.015 (3)	−0.003 (2)	0.018 (3)
C6	0.071 (3)	0.074 (4)	0.040 (3)	−0.006 (3)	0.002 (2)	0.009 (3)
C7	0.048 (3)	0.046 (3)	0.050 (3)	−0.006 (2)	−0.002 (2)	−0.005 (2)
C8	0.071 (3)	0.060 (3)	0.045 (3)	0.004 (3)	0.008 (2)	−0.015 (2)
C9	0.071 (3)	0.056 (3)	0.041 (3)	0.000 (3)	0.009 (2)	−0.002 (2)
C10	0.054 (3)	0.057 (3)	0.046 (3)	0.001 (2)	0.000 (2)	0.001 (2)
C11	0.041 (2)	0.044 (3)	0.049 (3)	0.005 (2)	0.001 (2)	0.005 (2)
C12	0.067 (3)	0.061 (4)	0.058 (3)	−0.002 (3)	0.010 (3)	0.008 (3)
C13	0.075 (4)	0.078 (4)	0.049 (3)	0.005 (3)	−0.001 (3)	−0.012 (3)
C14	0.069 (3)	0.067 (4)	0.041 (3)	0.011 (3)	−0.004 (2)	−0.013 (3)
C15	0.062 (3)	0.063 (3)	0.050 (3)	0.008 (3)	−0.005 (2)	−0.008 (3)
C16	0.042 (2)	0.049 (3)	0.043 (3)	0.013 (2)	0.0004 (19)	0.012 (2)
C17	0.072 (3)	0.038 (3)	0.056 (3)	−0.003 (2)	0.004 (2)	0.010 (2)
C18	0.073 (3)	0.043 (3)	0.050 (3)	0.002 (2)	−0.005 (2)	−0.007 (2)

### Geometric parameters (Å, °)

**Table:**

Zn—N1	2.062 (4)	C7—C8	1.388 (7)
Zn—N2	2.052 (4)	C8—C9	1.366 (7)
Zn—Cl1	2.2235 (13)	C8—H8	0.9300
Zn—Cl2	2.2262 (13)	C9—H9	0.9300
N1—C1	1.298 (6)	C10—C11	1.453 (6)
N1—C9	1.370 (6)	C10—H10	0.9300
N2—C10	1.290 (6)	C11—C12	1.381 (7)
N2—C18	1.379 (6)	C11—C16	1.409 (6)
C1—C2	1.441 (6)	C12—C13	1.363 (7)
C1—H1	0.9300	C12—H12	0.9300
C2—C3	1.385 (7)	C13—C14	1.387 (8)
C2—C7	1.403 (6)	C13—H13	0.9300
C3—C4	1.346 (7)	C14—C15	1.344 (7)
C3—H3	0.9300	C14—H14	0.9300
C4—C5	1.406 (8)	C15—C16	1.453 (7)
C4—H4	0.9300	C15—H15	0.9300
C5—C6	1.334 (8)	C16—C17	1.371 (6)
C5—H5	0.9300	C17—C18	1.359 (7)
C6—C7	1.450 (7)	C17—H17	0.9300
C6—H6	0.9300	C18—H18	0.9300
			
N2—Zn—N1	108.05 (16)	C9—C8—C7	117.9 (5)
N2—Zn—Cl1	106.47 (11)	C9—C8—H8	121.0
N1—Zn—Cl1	112.99 (10)	C7—C8—H8	121.0
N2—Zn—Cl2	108.96 (11)	C8—C9—N1	123.6 (5)
N1—Zn—Cl2	104.91 (11)	C8—C9—H9	118.2
Cl1—Zn—Cl2	115.26 (6)	N1—C9—H9	118.2
C1—N1—C9	118.1 (4)	N2—C10—C11	123.0 (5)
C1—N1—Zn	126.1 (3)	N2—C10—H10	118.5
C9—N1—Zn	115.8 (3)	C11—C10—H10	118.5
C10—N2—C18	118.7 (4)	C12—C11—C16	122.8 (5)
C10—N2—Zn	119.6 (4)	C12—C11—C10	121.6 (5)
C18—N2—Zn	121.6 (3)	C16—C11—C10	115.6 (4)
N1—C1—C2	123.9 (4)	C13—C12—C11	117.7 (5)
N1—C1—H1	118.1	C13—C12—H12	121.2
C2—C1—H1	118.1	C11—C12—H12	121.2
C3—C2—C7	121.8 (4)	C12—C13—C14	122.1 (5)
C3—C2—C1	122.6 (4)	C12—C13—H13	118.9
C7—C2—C1	115.6 (4)	C14—C13—H13	118.9
C4—C3—C2	118.4 (5)	C15—C14—C13	121.5 (5)
C4—C3—H3	120.8	C15—C14—H14	119.2
C2—C3—H3	120.8	C13—C14—H14	119.2
C3—C4—C5	122.1 (5)	C14—C15—C16	119.1 (5)
C3—C4—H4	118.9	C14—C15—H15	120.5
C5—C4—H4	118.9	C16—C15—H15	120.5
C6—C5—C4	120.8 (5)	C17—C16—C11	120.7 (5)
C6—C5—H5	119.6	C17—C16—C15	122.5 (5)
C4—C5—H5	119.6	C11—C16—C15	116.8 (4)
C5—C6—C7	119.3 (5)	C18—C17—C16	118.7 (5)
C5—C6—H6	120.3	C18—C17—H17	120.6
C7—C6—H6	120.3	C16—C17—H17	120.6
C8—C7—C2	120.8 (4)	C17—C18—N2	123.2 (5)
C8—C7—C6	121.7 (5)	C17—C18—H18	118.4
C2—C7—C6	117.5 (5)	N2—C18—H18	118.4

## References

[bb1] Altomare, A., Cascarano, G., Giacovazzo, C. & Guagliardi, A. (1993). *J. Appl. Cryst.* **26**, 343–350.

[bb2] Deisenhofer, J. & Michel, H. (1989). *EMBO J.* **8**, 2149–2170. 10.1002/j.1460-2075.1989.tb08338.x PMC401143 2676514

[bb3] Farrugia, L. J. (1997). *J. Appl. Cryst.* **30**, 565.

[bb4] Farrugia, L. J. (1999). *J. Appl. Cryst.* **32**, 837–838.

[bb5] Higashi, T. (1995). *ABSCOR* Rigaku Corporation, Tokyo, Japan.

[bb6] Li, M.-J., Nie, J.-J. & Xu, D.-J. (2010). *Acta Cryst.* E**66**, m840. 10.1107/S1600536810023895 PMC3006983 21587752

[bb7] Rigaku (1998). *PROCESS-AUTO* Rigaku Corporation, Tokyo, Japan.

[bb8] Rigaku/MSC (2002). *CrystalStructure* Rigaku/MSC, The Woodlands, Texas, USA.

[bb9] Sheldrick, G. M. (2008). *Acta Cryst.* A**64**, 112–122. 10.1107/S0108767307043930 18156677

[bb10] Spek, A. L. (2009). *Acta Cryst.* D**65**, 148–155. 10.1107/S090744490804362X PMC2631630 19171970

[bb11] Su, J.-R. & Xu, D.-J. (2004). *J. Coord. Chem.* **57**, 223–229.

[bb12] Xu, D.-J., Zhang, B.-Y., Su, J.-R. & Nie, J.-J. (2007). *Acta Cryst.* C**63**, m622–m624. 10.1107/S0108270107050597 18057612

